# Comparative transcriptome analysis of male and female flowers in *Spinacia oleracea* L

**DOI:** 10.1186/s12864-020-07277-4

**Published:** 2020-12-01

**Authors:** Ning Li, Ziwei Meng, Minjie Tao, Yueyuan Wang, Yulan Zhang, Shufen Li, Wujun Gao, Chuanliang Deng

**Affiliations:** College of Life Sciences, Henan Normal University, Xinxiang, 453007 China

**Keywords:** Spinach, Sex determination and differentiation, Gibberellin, Auxin, Sugar, Full-length transcriptome

## Abstract

**Background:**

Dioecious spinach (*Spinacia oleracea* L.), a commercial and nutritional vegetable crop, serves as a model for studying the mechanisms of sex determination and differentiation in plants. However, this mechanism is still unclear. Herein, based on PacBio Iso-seq and Illumina RNA-seq data, comparative transcriptome analysis of male and female flowers were performed to explore the sex differentiation mechanism in spinach.

**Results:**

Compared with published genome of spinach, 10,800 transcripts were newly annotated; alternative splicing, alternative polyadenylation and lncRNA were analyzed for the first time, increasing the diversity of spinach transcriptome. A total of 2965 differentially expressed genes were identified between female and male flowers at three early development stages. The differential expression of RNA splicing-related genes, polyadenylation-related genes and lncRNAs suggested the involvement of alternative splicing, alternative polyadenylation and lncRNA in sex differentiation. Moreover, 1946 male-biased genes and 961 female-biased genes were found and several candidate genes related to gender development were identified, providing new clues to reveal the mechanism of sex differentiation. In addition, weighted gene co-expression network analysis showed that auxin and gibberellin were the common crucial factors in regulating female or male flower development; however, the closely co-expressed genes of these two factors were different between male and female flower, which may result in spinach sex differentiation.

**Conclusions:**

In this study, 10,800 transcripts were newly annotated, and the alternative splicing, alternative polyadenylation and long-noncoding RNA were comprehensively analyzed for the first time in spinach, providing valuable information for functional genome study. Moreover, candidate genes related to gender development were identified, shedding new insight on studying the mechanism of sex determination and differentiation in plant.

**Supplementary Information:**

The online version contains supplementary material available at 10.1186/s12864-020-07277-4.

## Background

Most animals are dioecious, whereas only approximately 6% of angiosperm plants are dioecious [1]. Male and female sterile mutations in a pair of proto sex chromosomes result in dioecy production. To date, there are two viewpoints about sex determination mechanism in dioecious plants: one is two-gene mutant model and the other is single-factor mutant model. In two-gene mutant model, one mutant occurs in a stamen fertility-related gene and results in male sterility; the other mutant occurs in pistil-related gene and suppresses female organ development [2–7]. In single-factor mutant model, the emergence of dioecious plants may be caused by two independent mutations in a single gene, one is functional inactivation mutation leading to male sterility, and the other is functional acquisition mutation leading to female sterility [8, 9]. The genes mutated in the two theoretical models are called sex-determining genes, and the autosomes that carry the sex-determining genes evolved into sex chromosomes, and finally XY or ZW sex-determination system were formed [9]. Sex-determining gene is located in the non-recombining region of the Y/W chromosome, while, repetitive sequences are usually around the non-recombining region, which impedes the uncovering of sex determination mechanism in dioecious plants. Till now, only a few sex-determining genes have been reported in plants, such as *Diospyros lotus* [10, 11], *Asparagus officinalis* [12, 13], *Date palm* [14], *Actinidia chinensis* [15], *Vitis vinifera* [16, 17], *Ficus carica* [18], *Populus trichocarpa* [19] and *Fragaria octoploids* [20]. Although these identified genes are involved in or determine the emergence of unisexual flower, the exact mechanism of sex determination in plants remains unclear.

The differentiation of male and females is variable in dioecious plants. For example, the unisexual flower of some dioecious plants has both stamen primordia and pistil primordia and one is selectively aborted before maturity, such as garden asparagus. However, some other dioecious plants only form male or female primordia and then develop to male or female flower, such as spinach [21, 22]. Series of regulatory factors, such as transcription factors, noncoding RNAs, epigenetic marks, hormones and other metabolites are involved in sex differentiation [10, 21]. The sex-determining genes may cooperate with genetic networks and metabolic pathways involved in sex differentiation to control unisexual flower development. Hence, identification of the sex differentiation-related genes is helpful to elucidate the mechanism underlying the sex determination and differentiation.

Spinach (*Spinacia oleracea*, 2n = 12), an annual dioecious plant with short lifecycle, is a good model for studying sex determination of dioecious plants. Spinach is a member of *Spinacia* genus, which included another two species *S. turkestanica* Ilj. and *S. tetrandra* Stev.. The three species of the *Spinacia* genus are dioecious and occasionally monoecious, which is controlled by a pair of sex chromosomes XX/XY. The co-existence of homomorphic and heteromorphic sex chromosomes under genus *Spinacia* makes spinach as a good model plant for investigating the evolution of sex chromosomes. Researchers have exerted considerable effort to uncover the sex-determining region (SDR) in spinach. Many molecular markers were developed in spinach and serials of sex-linked molecular markers were identified, such as T11A, V20A, and SP_0018 [21–28]. 45 s rDNA can also be used to discriminate X and Y chromosome by FISH [29]. On the basis of sex-linked markers, Onodera and colleagues found a non-recombining region around the male-determining locus on the Y chromosome [30, 31]. These molecular markers are helpful in locating the accurate SDR and further identifying sex-determining genes. Qian et al. found the SDR located at 66.98–69.72 and 75.48–92.96 cM regions of the sex chromosome [32]. Spinach genome data was published in 2017 (http://www.spinachbase.org/) [33], and Chr4 is the sex chromosome where the sex-determining genes reside [27]. In 2020, an 18.4 Mb sex-linked region was released by Yu et al. [34]. These works provide valuable information for uncovering sex-determining genes. According to the classical ABC model for flower development, the B class floral identity genes *SpAPETALLA3* (*SpAP3*) and *SpPISTILLATA* (*SpPI*) have been identified as the masculinizing factors in spinach [35, 36]. Gibberellic acid (GA) induces sex conversion in spinach [37, 38]. Recently, a DELLA family gene, *GIBBERELLIC ACID INSENSITIVE* (*SpGAI*), has been characterized; it modulates GA level and further control the sex differentiation of spinach [39]. However, the mechanism of sex determination and differentiation in spinach remains unclear.

The length of a complete transcript is 1000–6000 bases in general. Due to the short reading length of the second generation sequencing technology, the resulting sequencing fragments need to be assembled, and the resulting transcripts may produce splicing errors and more chimeras, so that the complete transcripts cannot be obtained [40]. Pacbio Iso-seq, the third-generation sequencing technology with long reading length (average 15 KB), can help us directly obtained complete full-length transcripts without splicing. It is beneficial to improve the genome sequence and study mRNA structure such as alternative splicing, fusion gene and allele expression and so on [41].

Hence, we utilized PacBio Iso-seq and Illumina RNA-seq to detect the candidate genes for sex determination and differentiation. Weighted gene co-expression network analysis (WGCNA) was further performed to construct the regulation network of the candidate genes. Our study show that sex differentiation may be regulated by the crosstalk among sugar, auxin and gibberellin signaling; different transcription factors between female and male may receive signals and target cell development-related genes to modulate floral whorl initiation.

## Results

### PacBio Iso-seq sequencing

**Fig. 1:**
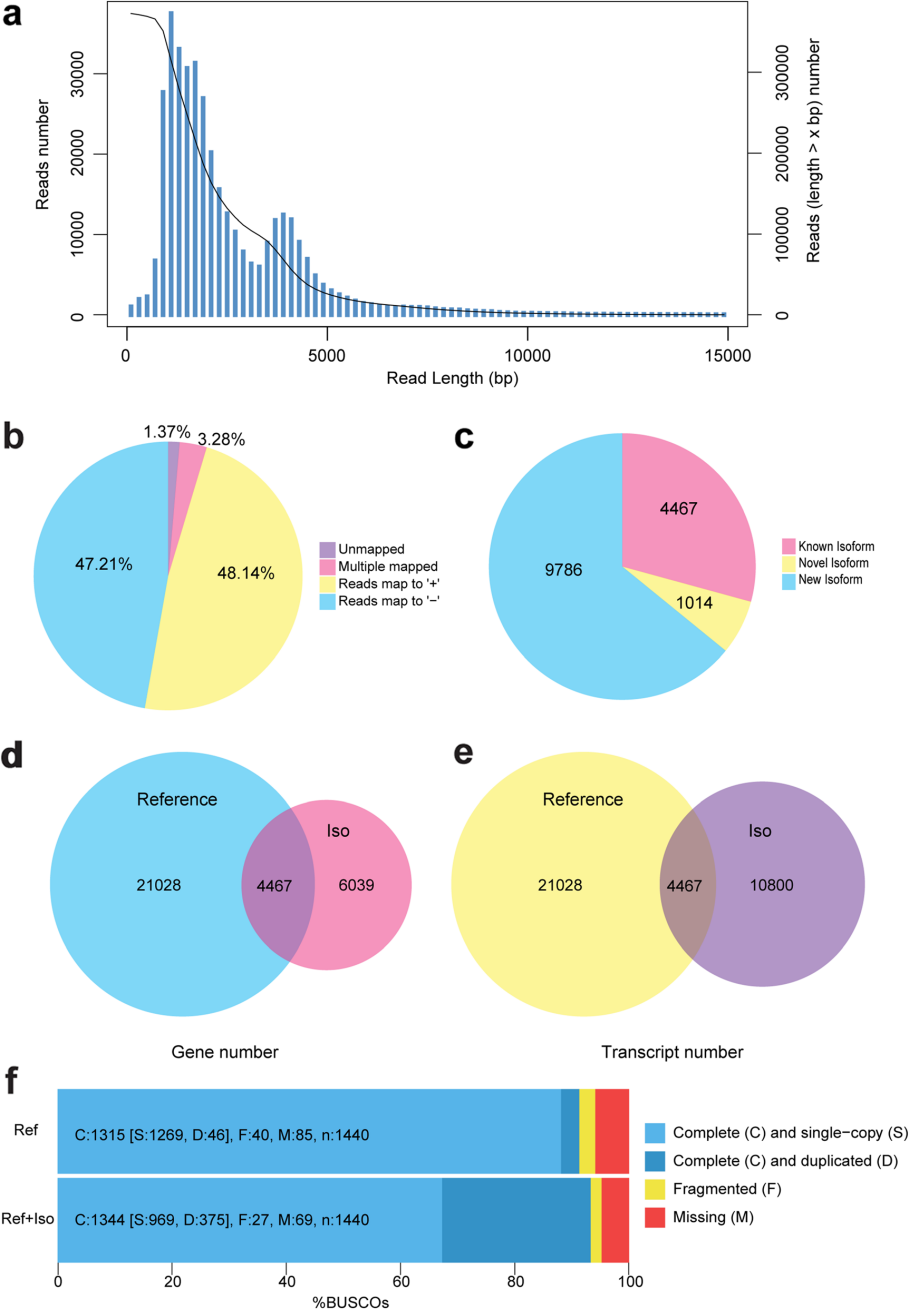
Summary of Iso-seq data. **a** CCS read length distribution, the X-axis represents the read length, the Y-axis on the left represents the column graph coordinate, indicating the number of reads with a certain length (X-axis), and the Y-axis on the right is the curve coordinate, indicating the number of reads whose length is bigger than a certain X-axis value. **b** The percentage of high quality isoforms mapped to the reference genome. **c** Classification and number of isoforms mapped to the reference genome. Known isoform was mapped to annotated genes in reference genome, new isoform was mapped to different exons of annotated gene in reference genome, and novel isoform was mapped to unannotated region in reference genome. **d** and **e** The number of genes and transcripts annotated in reference genome and Iso-seq data. **f** BUSCO assessment results of reference genome and optimized genome of spinach. Ref, reference genome; Ref + Iso, genome optimized via PacBio Iso-seq sequencing

The transcriptome of the spinach flower at the early developmental stage were sequenced using the PacBio Iso-seq platform (Additional file 1). A total of 373,155 circular consensus sequence reads were obtained. The reads length ranged from 50 bp to 15,000 bp, and the mean value was 2483 bp (Fig. 1a). We obtained 34,443 high-quality isoforms, of which 98.63% were mapped to the reference genome (Fig. 1b). A total of 15,267 isoforms encoded by 10,506 genes were mapped to the reference genome after clustering and removing redundancy. Among these mapped isoforms, 4467 known isoforms (mapped to 4445 annotated genes), 9786 new isoforms (mapped to the different exons of annotated gene), and 1014 novel isoforms (mapped to unannotated region of the genome) were found (Fig. 1c). The isoforms were annotated with the NCBI non-redundant protein (Nr), Kyoto Encyclopedia of Genes and Genomes (KEGG), Swiss-Prot, and Gene Ontology (GO) databases. Conclusively, 6039 new genes can be added to the reference genome (Fig. 1d). Moreover, 10,800 transcripts encoded by 6061 genes were newly annotated, thereby increasing the diversity of the reference transcriptome (Fig. 1e). Moreover, we evaluated the BUSCO score of spinach genome optimized by our full length transcriptome, which is 93.33% and higher than published spinach genome (91.32%). Clearly, our work improved the spinach genome annotation (Fig. 1f).

### The genes residing in sex chromosome

**Fig. 2:**
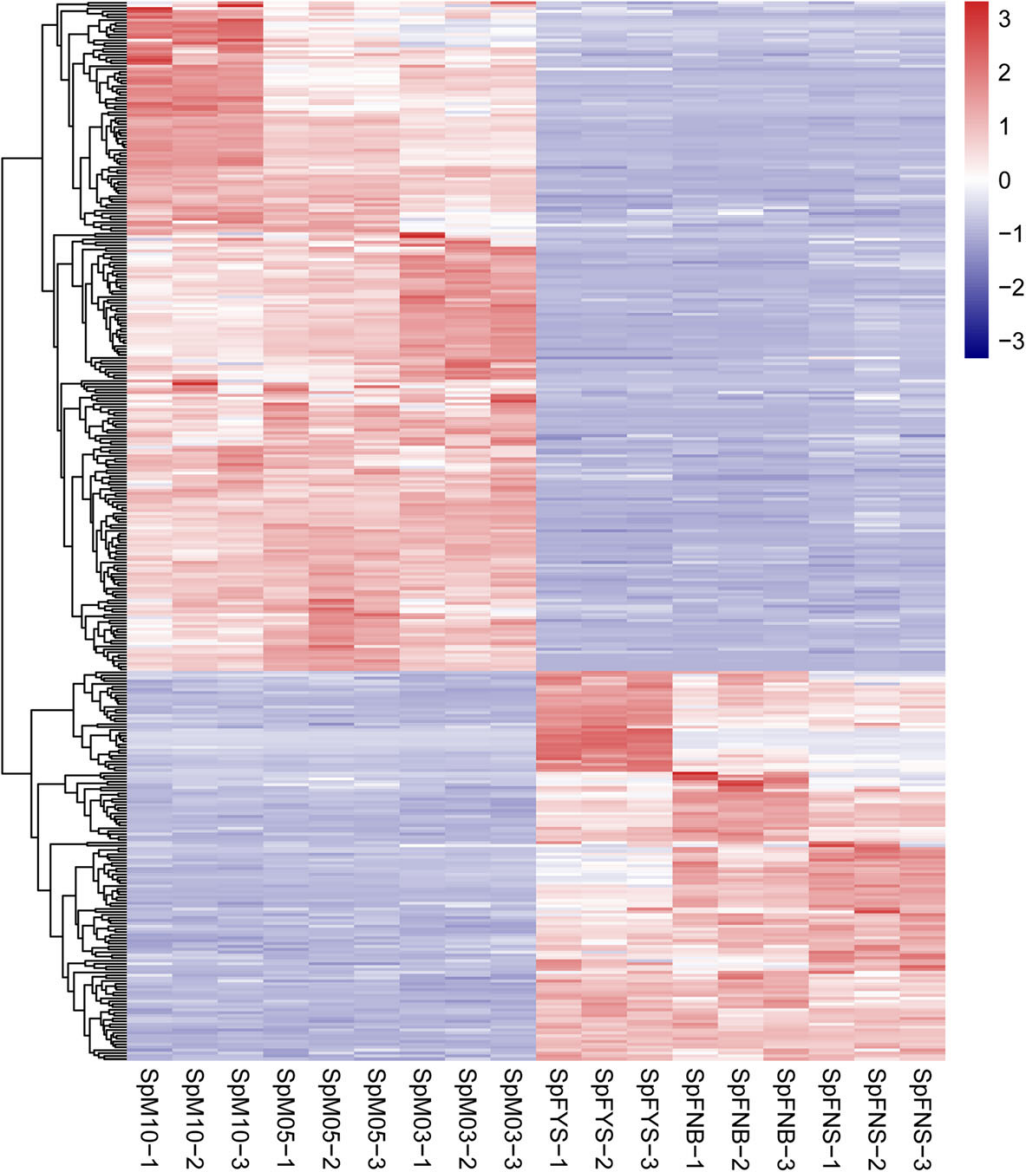
Expression pattern clustering analysis of sex-biased genes residing in sex chromosome

In the published spinach genome data, chr4 is the sex chromosome where the sex-determining genes reside [27]. According to Iso-seq data, 2190 isoforms encoded by 1489 genes reside in chr4. Blasting with spinach genome, we found 80 isoforms are single with no copy in other chromosomes or scaffolds, and the rest isoforms are duplicated. The expression of these isoforms residing in sex chromosome was further analyzed using RNA-seq data and 367 isoforms showed sex-biased expression (Fig. 2). The sex determining region (SDR) has not been fully uncovered in spinach. Till now, at least 12 genes in male-specific region were identified by Okazaki et al. (2019) [27] and a 18.4 Mb sex-linked region was revealed by Yu et al. (2020) [34]. Blasting with these genes or sequence, ten sex-biased genes fall within the 18.4 Mb sex-linked regions, which may be involved in sex determination or differentiation in spinach (Table 1).

**Table 1:** List of sex-biased genes residing in 18.4 Mb of sex-linked region in spinach

Gene ID	Genome location	Type	Log_**2**_(Fold Change)			Description
			M03/FNS	M05/FNB	M10/FYS	
*Gene003616*	chr4: (+) 91,520 … 105,982 bp	new	−1.9	− 1.66	− 1.07	cyclin-dependent kinase
*Gene004137*	chr4: (−) 50,043,916 … 50,045,261 bp	novel	1.36	1.38	1.49	uncharacterized LOC104902451
*Gene004138*	chr4: (+) 50,048,233 … 50,049,888 bp	novel	3.35	4.75	4.9	serine/threonine-protein kinase D6PKL1-like
*Gene004422*	chr4: (−) 90,648,657 … 906,531,003 bp	novel	−1.67	−1.73	−2.54	uncharacterized LOC104906114
*Gene005061*	chr4: (+) 122,096,317 … 122,097,168 bp	novel	7.12	F = 0	F = 0	–
*Spo16245*	chr4: (−) 57,224,763 … 57,239,708 bp	known	F = 0	F = 0	F = 0	RNA-binding family protein
*Spo21056*	chr4: (+) 50,045,074 … 50,047,854 bp	known	1.07	1.79	1.8	Microtubule associated family protein
*Spo26984*	chr4: (−) 41,263,089 … 41,268,484 bp	known	3.34	3.96	3.1	ABC transporter B family member 11
*XLOC_021290*	chr4: (+) 57,293,175 … 57,294,874 bp	new	−4.24	−3.1	−3.86	–
*XLOC_021752*	chr4: (+) 99,677,867 … 99,680,635 bp	new	4.98	F = 0	F = 0	–

Note: “F = 0” means the gene expression is not detected in female sample

SNP and InDel analysis were performed for RNA-seq data. We identified 131 SNPs in 114 sex-chromosomal genes, which were homozygous mutations (1|1) or no mutations (0|0) in all female samples and heterozygous mutations (0|1) in all male samples. Moreover, 11 of the 114 sex-chromosomal genes displayed sex-biased expression (Additional file 10). Of the 114 sex-chromosomal genes, two genes overlap with the Y-linked genes [27] and one gene falls within 18.4 Mb sex-linked region [34], but their expression were not sex-biased (Additional file 10).

### Alternative splicing analysis

**Fig. 3:**
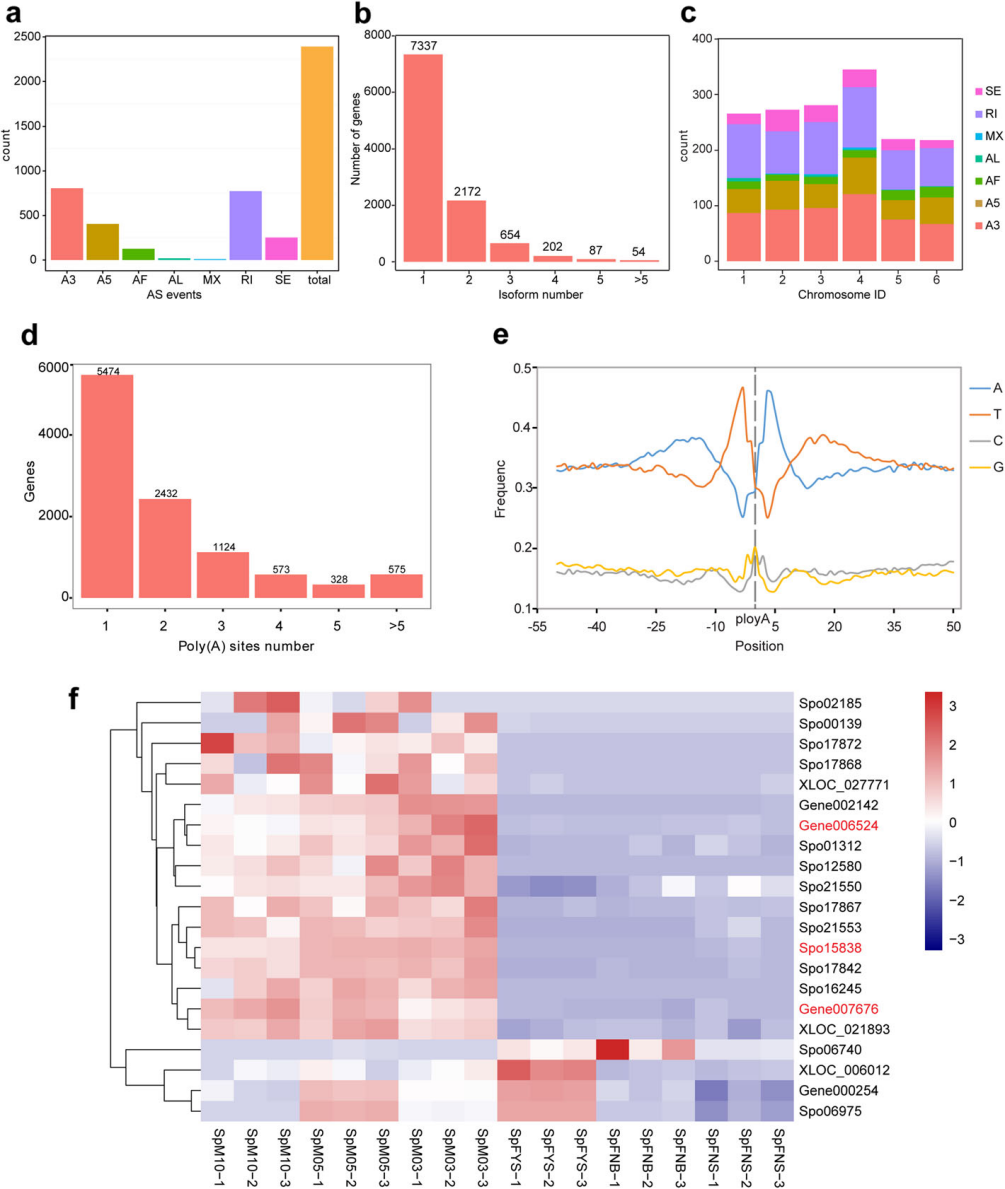
Summary of alternative splicing events and alternative polyadenylation events. **a** Alternative splicing events classification and number statistics. AS, alternative splicing; SE, skipping exon events; A5, 5′ splice sites events; A3, 3′ splice sites events; MX, mutually exclusive exons events; RI, retained intron events; AF, alternative first exons events; AL, alternative last exons events. **b** Distribution of genes that produce one or more splice isoforms. **c** Distribution of alternative splicing events in assembled chromosomes. **d** Distribution of the number of poly(A) sites per gene. **e** Relative frequency of each nucleotide around poly(A) cleavage sites. **f** The differentially expressed genes related to alternative splicing and alternative polyadenylation, the genes in red font are related to alternative polyadenylation, the genes in black font are related to alternative splicing

Alternative splicing is an important mechanism of post-transcriptional regulation and influences protein coding to modulate plant development. Using the Iso-seq data, we investigated alternative splicing in spinach flower for the first time. There are 2391 alternative splicing events, including 252 skipping exon (SE), 405 alternative 5′ splice sites (A5), 805 alternative 3′ splice sites (A3), 10 mutually exclusive exons (MX), 773 retained intron (RI), 126 alternative first exons (AF), and 20 alternative last exons (AL) events (Fig. 3a). As shown in Fig. 3b, 7,337 genes (69.84%) generated only a single isoform, 3169 genes (30.16%) produced two to five transcripts, and 54 genes encoded 6–14 splice isoforms. Furthermore, 8442 annotated genes in the reference genome yielded splicing variants in our data. Five genes were randomly selected to validate the alternative splicing events by RT-PCR (Additional file 2). We analyzed the distribution of alternative splicing events in the six assembled chromosomes and found that the sex chromosome chr4 produced the most alternative splicing events (Fig. 3c). There are 1489 genes reside in chr4, 199 of which produced alternative splicing isoforms. Through alternative splicing, a gene may produce variant transcripts to regulate different biological process, such as induce of female or male floral primordia. According to RNA-seq results, 18 genes related to RNA splicing displayed differentially expressed and 15 of them showed sex-biased expression (described later) (Fig. 3f), indicating that alternative splicing may be involved in spinach sex differentiation.

### Alternative polyadenylation analysis

Most eukaryotic genes contain more than one polyadenylation sites. Alternative polyadenylation influence the coding region, stability, and localization and translation efficiency of mRNA [42]. Alternative polyadenylation can increase the complexity of the transcriptome and regulate gene expression to control various plant developmental processes from cell cycle to flowering and floral organ identity [43, 44].

Herein, alternative polyadenylation analysis in spinach flower was first performed using Iso-seq. Of the 10,506 genes obtained by Iso-seq, 5032 have more than two alternative polyadenylation sites (Fig. 3d). Five genes were randomly selected to verify the alternative polyadenylation events by 3′ rapid amplification of cDNA ends (3′ RACE) (Additional file 2). The clear bias of nucleotides was determined by analyzing the nucleotide composition upstream (− 50 nts) and downstream (+ 50 nts) of polyadenylation sites, as shown in Fig. 3e. The nucleotide bias is similar to 3′ UTR profiles as previously reported [45]. According to RNA-seq results, three genes related to polyadenylation showed sex-biased expression (described later) (Fig. 3f). Hence, alternative polyadenylation occurring in flower development-related genes may be a crucial regulatory mechanism of sex differentiation.

### LncRNA identification

**Fig. 4:**
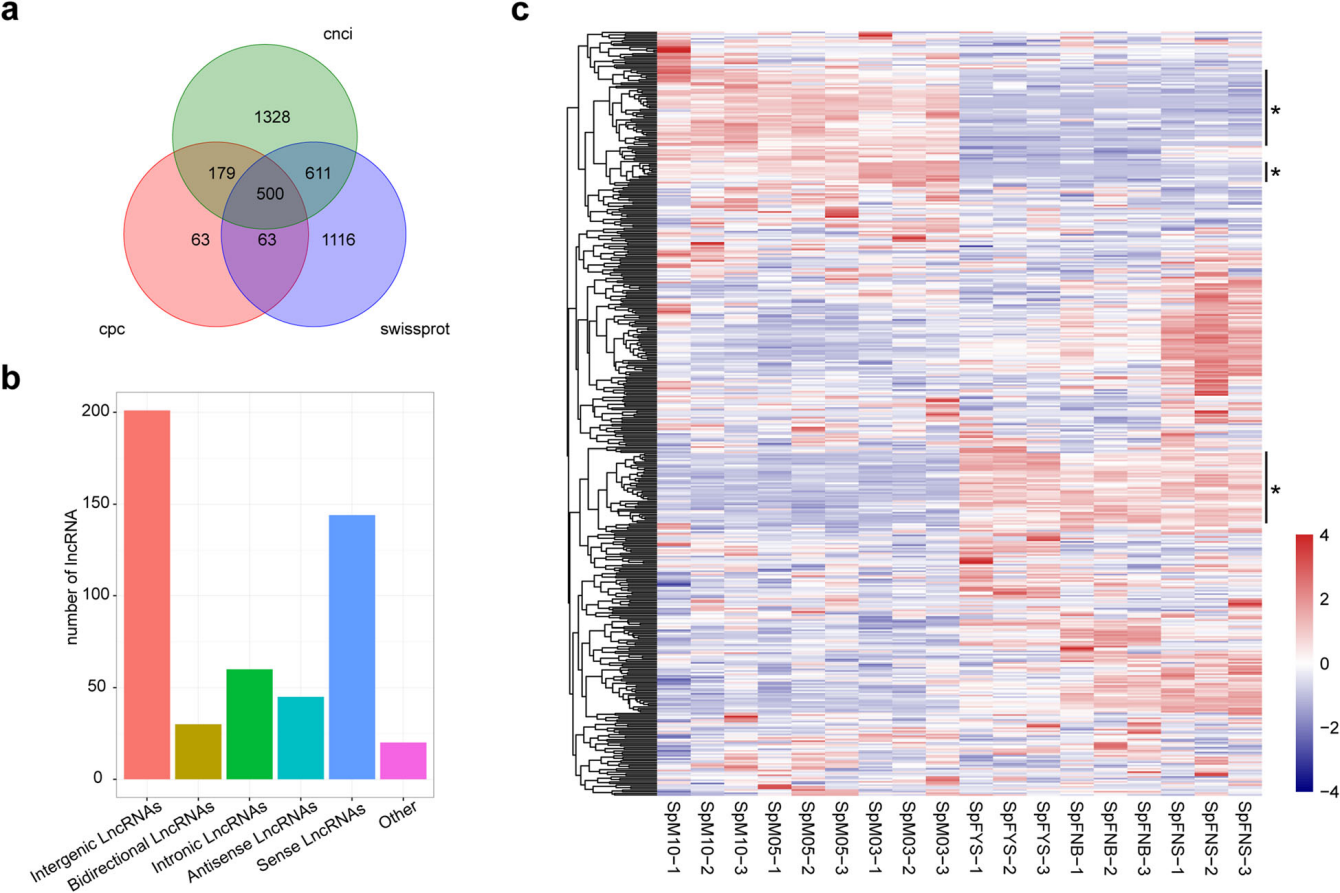
LncRNA analysis. **a** The number of lncRNA identified by CPC, CNCI and SwissProt. **b** Distribution of each type of lncRNA. **c** Expression pattern clustering analysis of lncRNAs in female and male flower; each column represents a sample, each sample has three replicates; each row represents a gene; sex-biased expression lncRNAs were marked with asterisk and vertical line

Long non-coding RNAs (lncRNAs) can participate in eukaryotic gene regulation. However, lncRNAs are still largely unknown in spinach, and herein lncRNAs were analyzed in spinach for the first time. After assessing the protein-coding potential with CPC and CNCI software and annotating protein with Swiss-Prot database, we identified 500 lncRNAs, which include 201 intergenic lncRNAs, 30 bidirectional lncRNAs, 61 intronic lncRNAs, 45 antisense lncRNAs, 149 sense lncRNAs, and 20 other lncRNAs (Fig. 4a, b). Five genes were randomly selected to verify the lncRNAs by PCR (Additional file 3). As shown in Fig. 4c, the expression profile of 42 lncRNAs (marked by asterisk) is distinct between male and female flowers, confirming the tissue-specific expression of lncRNA. The distinct expression of four lncRNAs (Gene004422, Gene002735, Gene004186 and Gene001186) was further validated by qPCR (Additional file 8). Hence, lncRNAs with sex-biased expression (described later) may be involved in the sex differentiation.

### Sex-biased genes of spinach

**Fig. 5:**
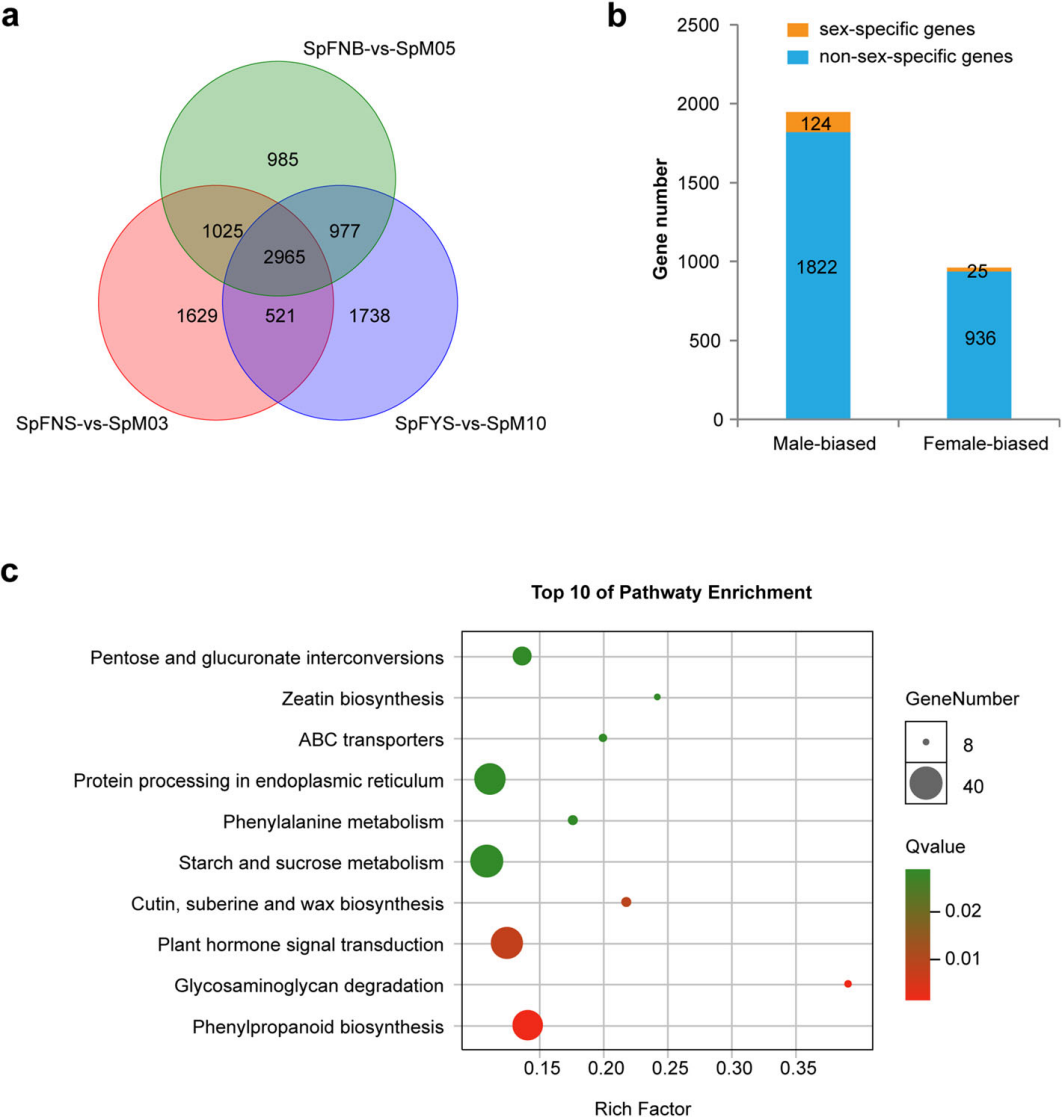
Analysis of DEGs between female and male flower. **a** The Venn diagram of DEGs among three flower stages. **b** The male- and female-biased genes number, orange bar indicates sex-specific genes number. **c** The top ten enriched KEGG pathways of sex-biased genes

Sex-biased genes, which exhibit significantly higher expression in one sex than in the other sex, always act downstream of sex-determining gene. Hence, identification of the sex-biased genes is helpful to uncover the sex-determination mechanism [46]. Herein, a total of 2965 DEGs (differentially expressed genes) were identified between female and male flowers at three early developmental stages (Fig. 5a). Of the DEGs, 1946 genes are male-biased genes and 961 genes are female-biased genes (Fig. 5b). Moreover, there are 124 male-specific genes and 25 female-specific genes (Fig. 5b). Through GO functional enrichment analysis of these DEGs, we found that the end term of Directed Acyclic Graph is sugar transmembrane transporter activity (GO:0051119) in molecular function and carbohydrate transport (GO:0008643) in biological processes (Additional file 4). Moreover, these genes were obviously enriched in 11 pathways via KEGG analysis. The top 5 pathways are glycosaminoglycan degradation (ko00531), protein processing in endoplasmic reticulum (ko04141), phenylpropanoid biosynthesis (ko00940), pentose and glucuronate interconversions (ko00040), and plant hormone signal transduction (ko04075) (Additional file 4).

**Fig. 6:**
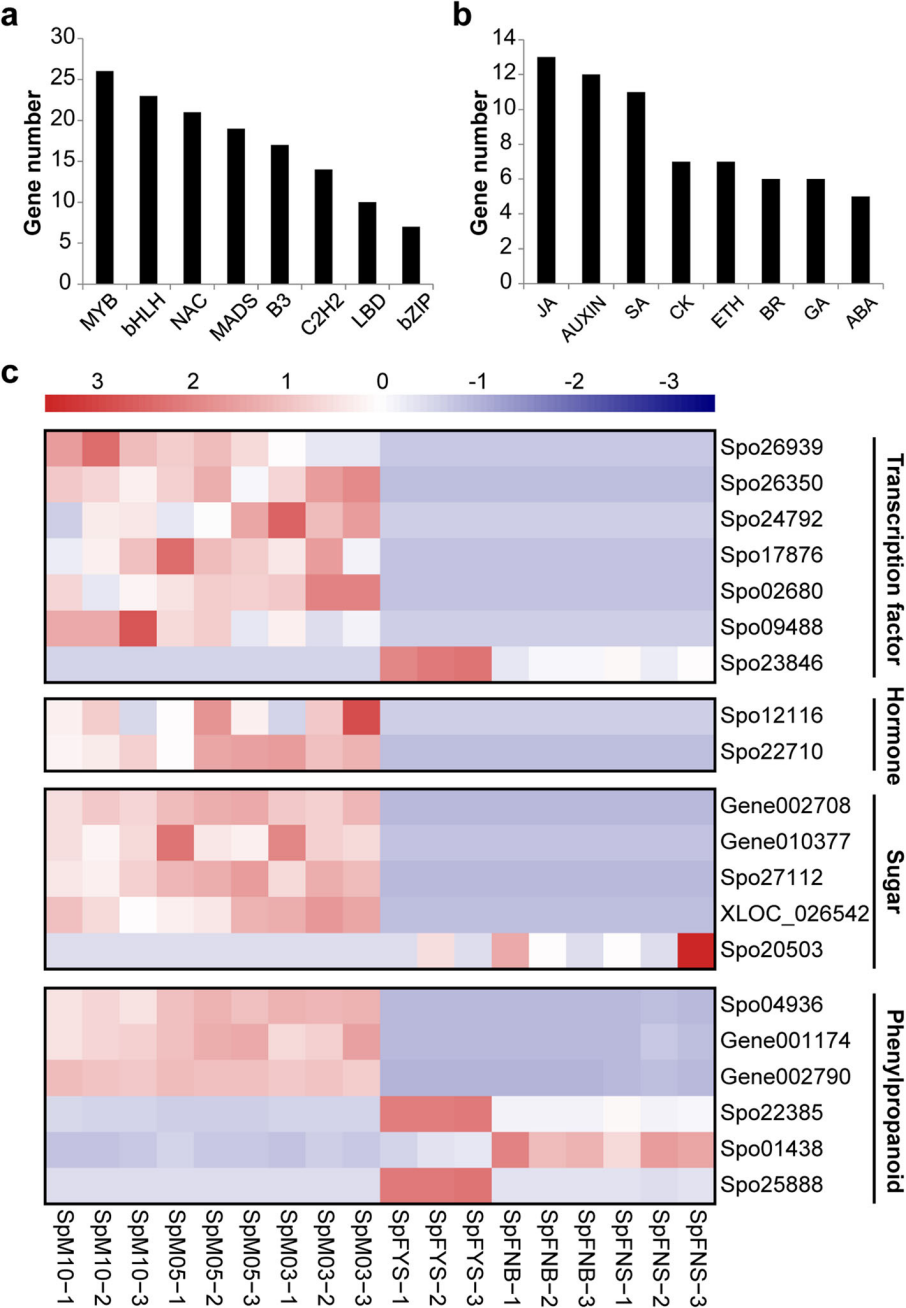
Analysis of sex-biased genes. **a** Statistics of sex-biased genes related to transcription factors (top eight families). **b** Statistics of sex-biased genes related to hormones. **c** Heat map of sex-specific DEGs related to transcription factors, hormones and sugar and top six sex-biased DEGs related to phenylpropanoid

Among the DEGs, 206 transcription factors were differentially expressed between female and male flowers at the three early developmental stages. These genes are from 30 transcription factor families, of which MYB (including 26 DEGs), bHLH (including 23 DEGs), NAC (including 21 DEGs) and MADS-box (including 19 DEGs) family are the predominant transcription factor families (Fig. 6a). Of these differentially expressed transcription factors, 111 genes exhibited male-biased expression and 95 genes showed female-biased expression (Additional file 5). Moreover, six transcription factors, including two NAC genes (*Spo02680* and *Spo17876*), two bHLH genes (*Spo26350* and *Spo26939*), one WRKY gene (*Spo09488*) and one C3H gene (*Spo24792*), show male-specific expression; and only one C2H2 gene (*Spo23846*) exhibits female-specific expression (Fig. 6c).

There were 67 DEGs involved in hormone pathway; of these DEGs, 13 were involved in JA pathway, 12 were involved in auxin pathway, 11 were involved in SA pathway, seven were involved in CK pathway, seven were involved in ethylene pathway, six were involved in gibberellin pathway, five were involved in BR pathway, and five were involved in ABA pathway (Fig. 6b). All these 67 hormone-related DEGs displayed sex-biased expression; 33 genes showed female-biased expression, while 34 genes exhibited male-biased expression. Moreover, we found that two auxin-related genes, *Spo12116* and *Spo22710*, displayed male-specific expression (Fig. 6c, Additional file 5).

### Co-expression networks of female and male flower

**Fig. 7:**
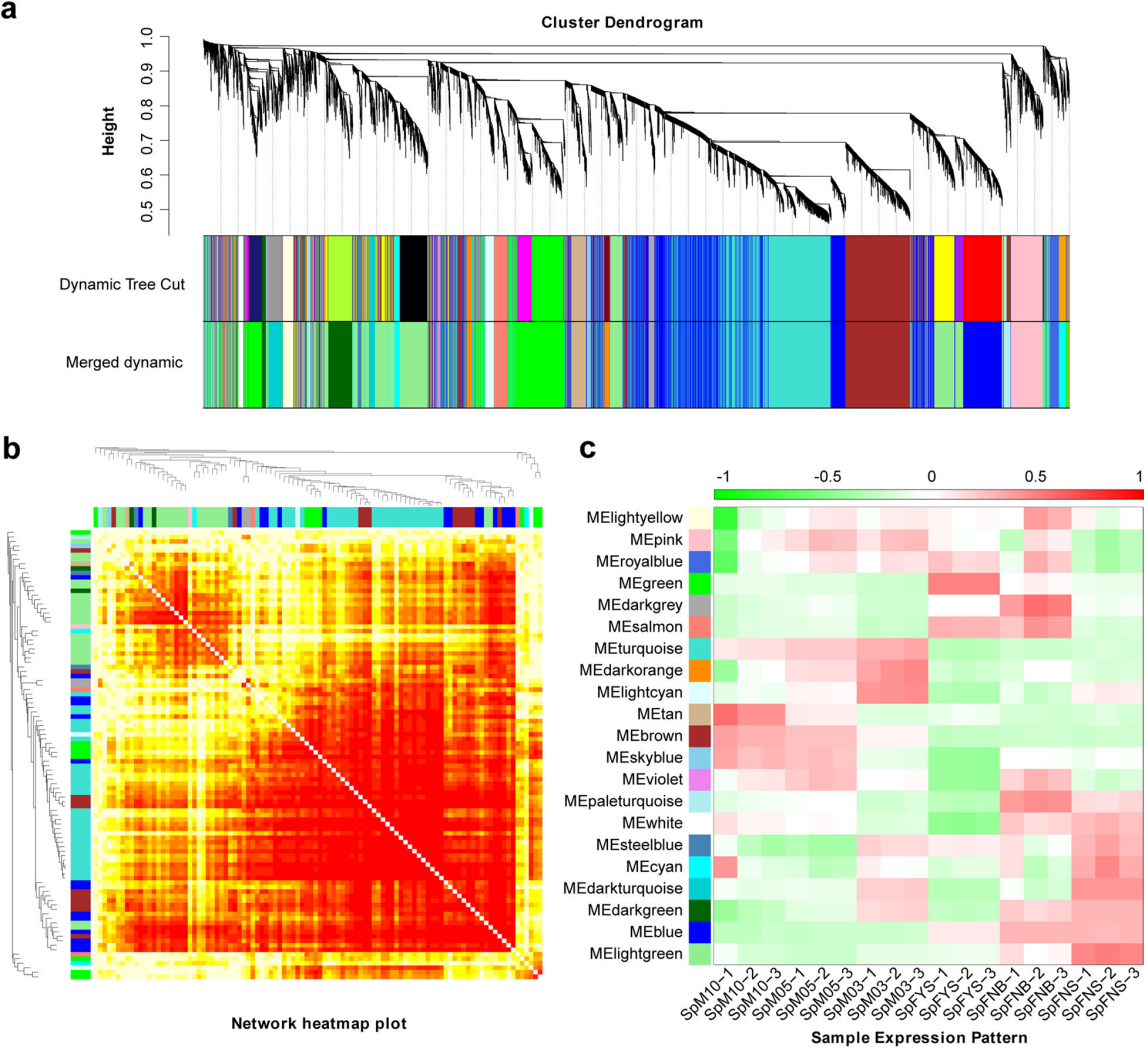
Weighted gene co-expression network analysis. **a** Hierarchical cluster tree showing co-expression modules identified using WGCNA; “height” represents the distance between two nodes (between genes), and the transverse distance is meaningless; “Dynamic Tree Cut” represents modules divided according to clustering results, “Merged dynamic” represents the merge of the module with similar expression pattern according to the module similarity, and the analysis is conducted according to the merged module. **b** Module gene correlation analysis; each row and column represents a gene, and the darker the color of each point (white → yellow → red) means higher connectivity between the two genes. **c** Sample expression pattern analysis; the abscissa represents the sample, the ordinate represents the module, and the expression patterns of module genes in each sample were displayed by module eigenvalues

**Fig. 8:**
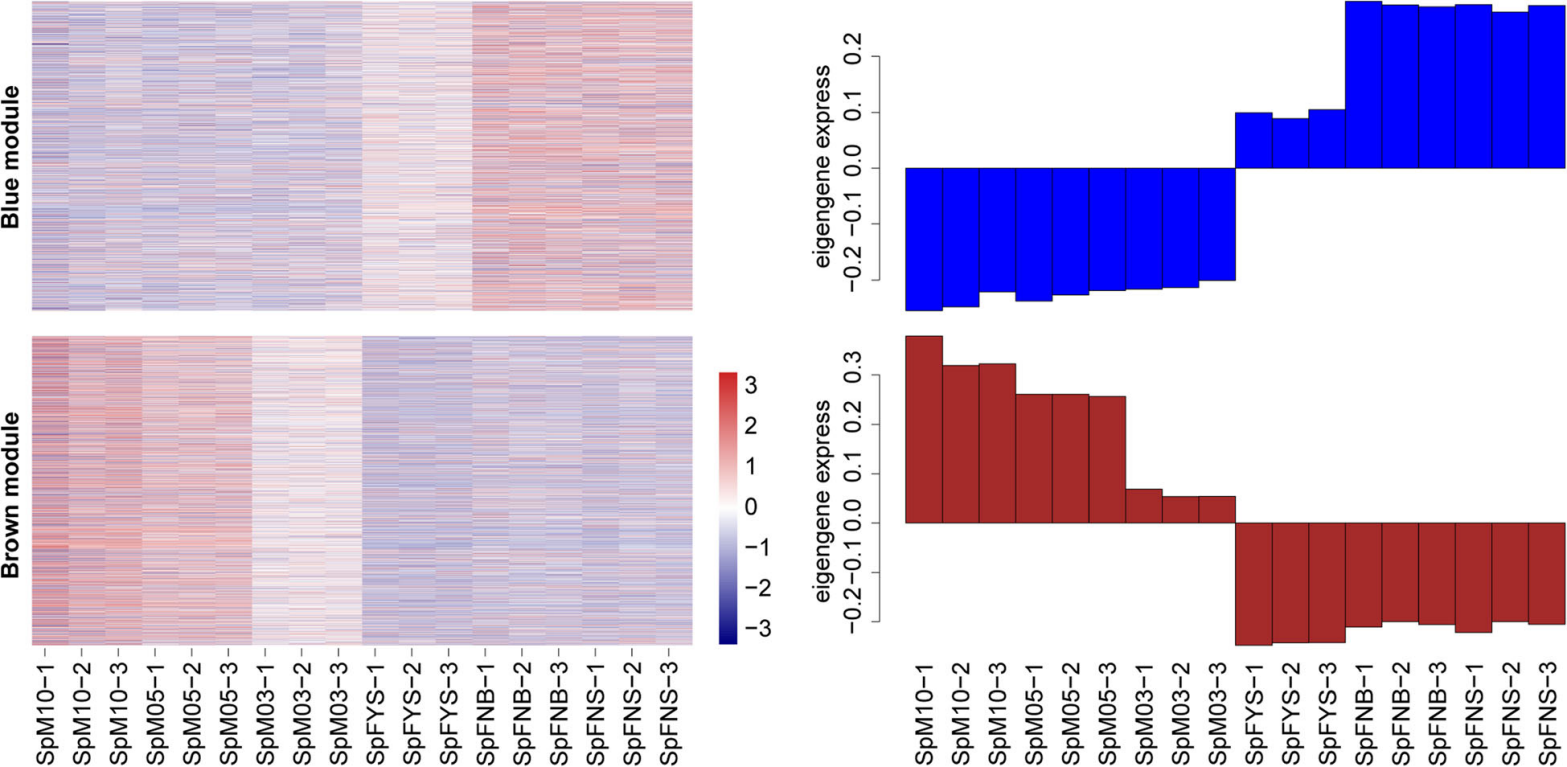
Expression of sex-biased expression modules. The heat map on left showed the expression of genes in the module in different samples, with the red being up-regulated and the blue being down-regulated; the bar chart on right shows the expression pattern of module eigenvalues in different samples

We performed weighted gene co-expression network analysis (WGCNA) to construct a potential regulatory network of sex differentiation in spinach. As shown in Fig. 7a, 16 modules were established by WGCNA in 18 samples including male and female flowers at different stages. These genes are naturally clustered according to the sexuality and developmental stage. As shown in Fig. 7b, the connectivity between each two genes was calculated, and the genes in the same module displayed strong connectivity. The expression pattern of each module was analyzed in all samples (Fig. 7c). Notably, the eigengene expression level of the blue module is female-biased, whereas that of the brown module is male-biased (Figs. 7c; 8); and these two modules were closely negatively correlated (Additional file 6, R = − 0.90, *p* = 5.17E-07). We defined the blue module as female module and brown module as male module.

**Fig. 9:**
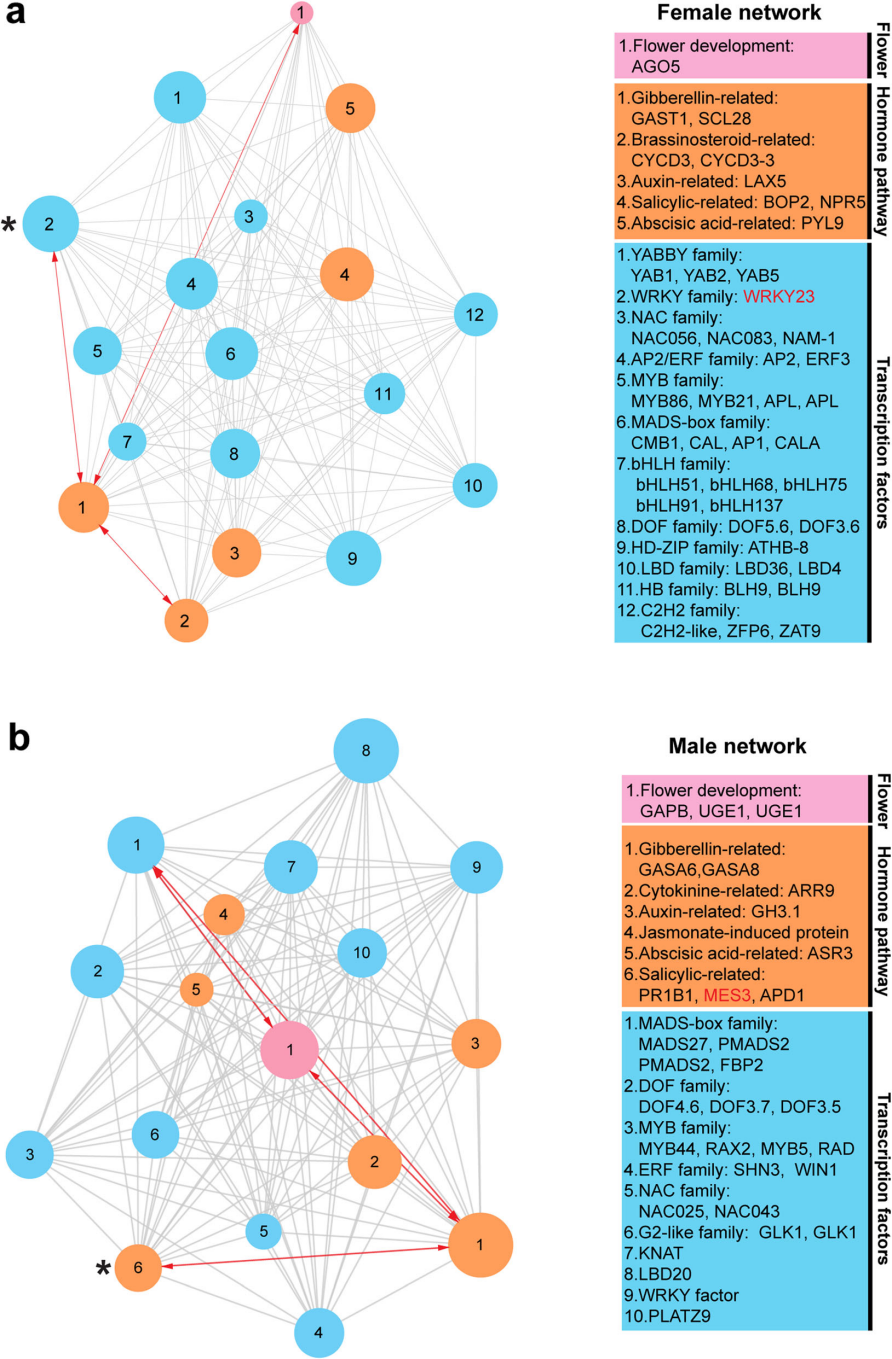
Visualization of female (**a**) and male (**b**) co-expression network. In the left network, the black stars marked hub gene, pink circles indicated flower-related genes, orange circles indicated hormone-regulated genes, blue circles indicated transcription factors, and the bigger the size of the circle means higher connectivity; the red line with arrow linked two most closely correlated genes. In the right box, genes involved in the network were listed and hub genes were in red font; gene in each circle can be found in the right box according to the color and Arabic numeral of the circle

To construct the co-expression network of female module and male module, we screened the differentially expressed genes related to flower development and hormone and differentially expressed transcription factors from female and male module. As a result, 41 DEGs were selected from female module, and 37 DEGs were selected from male module (Additional file 7). As shown in Fig. 9a, the hub gene of the female network is *Gene005140*, encoding a WRKY transcription factor. In the female network, the most closely correlated gene of the hub gene is *Spo24877* (weight value is 0.4899), encoding a Gibberellin-regulated protein and homologous to *AtGASA13* (*AT3G10185*); *Gene006949* (encoding cyclin D3 protein) is the most closely correlated gene of *Spo24877* (weight value is 0.4951) (Table 2; Fig. 9a). Moreover, eight genes related to gibberellin, auxin, abscisic acid, brassinosteroid, and salicylic pathway were involved in female network; 32 transcription factors from 12 families were found in female network; additionally, there is one floral organ development-related gene, *Spo10951*, encoding an argonaute protein and homologous to *AtAGO5* (*AT2G27880*) (Fig. 9a).

**Table 2:** Hub gene in female and male network

Network	ID	Connectivity	Symbol	Arabidopsis homolog	Weight value
**Female**	^a^Gene005140	1217.92	WRKY23	AT2G47260	
	^b^Spo24877	1164.75	GAST1	AT3G10185	^b^0.4899
	^c^Gene006949	1196.59	CYCD3–3	AT3G50070	^c^0.4951
**Male**	^d^Gene000891	903.44	MES3	AT2G23610	
	^e^Spo08103	803.60	GASA6	AT1G74670	^e^0.5074
	^f^Gene005514	723.19	FBP2	AT5G15800	^f^0.5121

Note: “a” and “d” indicated the hub gene of female and male network, respectively; “b” and “e” indicated the most closely co-expressed gene of “a” and “d” gene, respectively, and the weight value between them; “c” and “f” indicated the most closely co-expressed gene of “b” and “e” gene, respectively, and the weight value between them

As shown in Fig. 9b, the hub gene of the male network is *Gene000891*, encoding salicylic acid-binding protein 2-like and homologous to *AtMES3* (*AT2G23610*). In this male network, the most closely correlated gene of the hub gene is *Spo08103* (weight value is 0.5074), encoding a Gibberellin-regulated protein *GASA6* and homologous to *AtGASA6*; the most closely correlated gene of *Spo08103* is *Gene005514* (weight value is 0.5121), encoding a MADS-box protein and homologous to *AtSEP1* (Table 2; Fig. 9b). Moreover, nine genes related to gibberellin, auxin, abscisic acid, salicylic, jasmonate and cytokinine pathway were involved in male network; 24 transcription factors from 10 families were found in male network; additionally, there are three floral development-related genes, including two UDP-glucose 4-epimerase family proteins (*Spo00735* and *Spo00708*) and one Glyceraldehyde-3-phosphate dehydrogenase (*Spo21495*) (Fig. 9b).

## Discussion

### Full-length transcriptome analysis

For the first time, we comprehensively analyzed the full-length transcriptome in spinach. In summary, Iso-seq data yielded a number of novel findings, including (i) identification of transcriptome-wide full-length isoforms in spinach at an unprecedented scale with 15,267 isoforms (including 10,800 newly annotated transcripts), (ii) detection of 2391 splicing events compared with the reference genome for the first time, (iii) discovery of extensive alternative polyadenylation of spinach transcripts (47.90% of expressed genes have multiple polyadenylation sites); and (iv) identification of 500 newly annotated lncRNAs in spinach for the first time. These newly annotated transcripts and analysis of alternative splice isoforms and lncRNA would provide valuable resource to improve spinach genome annotation and for future functional studies.

Alternative splicing is an important post-transcriptional regulation mechanism that affects protein coding to modulate plant development. Alternative splicing is sensitive to inner and outer cues, such as hormone and biotic and abiotic factors, affecting plant growth [47–51]. In the current study, 30.26% genes had more than two alternative splicing isoforms. Alternative polyadenylation influences the coding region, stability, and localization and translation efficiency of mRNA [42]. Thus, it can regulate gene expression to control various plant developmental processes, such as floral organ identity [43, 44, 52–54]. Most of the DEGs associated with RNA splicing and polyadenylation showed sex-biased expression (Fig. 3). Hence, alternative splicing and alternative polyadenylation may play roles in sex differentiation. LncRNAs play roles in regulation of transcription, splicing, and nuclear structure and influences flowering time, root organogenesis, and reproduction [55–57]. As shown in Fig. 4c, 42 lncRNAs (marked by asterisks) showed distinct expression between male and female flowers, indicating their involvement in the regulation network of sex differentiation. The analysis of alternative splicing, alternative polyadenylation, and lncRNA revealed that various post-transcriptional regulations were involved in the sex differentiation and provided more clues to explore the mechanism of sex determination and differentiation.

### Candidate genes for spinach sex determination or differentiation

Sex-determining gene always locates in sex chromosome. In this study, 367 of 1489 sex-chromosomal genes showed sex-biased expression (Fig. 2). Moreover, ten of the 367 sex-biased genes fall within the 18.4 Mb sex-linked region [34], which can serve as candidates for sex determination or sex differentiation (Table 1). In addition, 114 sex-chromosomal genes identified by SNP analysis may be sex-linked genes, including 11 sex-biased genes; interestingly, two genes of them overlap with the reported Y-linked genes [27] and one gene falls within 18.4 Mb sex-linked region [34], but they were not sex-biased genes (Additional file 10). These candidate genes need to be validated in future work.

Identification of the sex-biased genes related to flower development is helpful for uncovering the sex determination and differentiation mechanism. Herein, 2907 sex-biased genes were found during the three early flower development stages, including 1946 male-biased genes and 961 female-biased genes; moreover, there are 124 male-specific genes and 25 female-specific genes (Fig. 5b). GO and KEGG analysis indicated that these sex-biased genes were predominantly functionally enriched in sugar metabolism, phenylpropanoid biosynthesis, and plant hormone signal transduction (Fig. 5c).

Sugars generated by photosynthesis and carbon metabolism in source and sink tissues can act as a signal to modulate various processes, such as photosynthesis, nutrient mobilization, and allocation, finally stimulating sink tissue growth, such as flower initiation and floral organ development [58, 59]. Hexokinase (HXK) and trehalose pathways are well defined in relation to sugar signaling. For example, HXK-involved sugar signaling pathway affects monoecious cucumber flower development, that is, femaleness process [60]. In maize, a trehalose-6-phosphate phosphatase, RAMOSA3, controls inflorescence architecture by modifying trehalose-dependent sugar signaling [61]. In this study, we found 88 sex-biased genes related to sugar metabolism; of them, one gene exhibited female-specific expression (*Spo20503*) and four genes exhibited male-specific expression (*Gene002708*, *Gene010377*, *Spo27112* and *XLOC_026542*) (Fig. 6, Additional file 5); the roles of these genes in spinach sex differentiation will be further studied.

Phenylpropanoid and downstream flavonoid and anthocyanin pathway are involved in male fertility [62–67]. We found 43 phenylpropanoid-related genes showed sex-biased expression (Additional file 5), encoding 4-coumarate--CoA ligase (4CL), beta-glucosidase (BGLU), caffeic acid 3-O-methyltransferase (COMT), caffeoyl-CoA O-methyltransferase (CCoAOMT), chalcone synthase (CHS), cinnamyl-alcohol dehydrogenase (CAD), cytochrome P450 protein (CYP450), dihydroflavonol 4-reductase (DR), flavanone-3-hydroxylase (F3H) and peroxidase (PER). These genes are intriguing and need to be validated in future work.

Phytohormones are important regulators of flower development, such as auxin, ethylene, gibberellin (GA), cytokinin (CK), abscisic acid (ABA), brassinosteroid (BR) and jasmonate (JA) [68–70]. One hormone may crosstalk with other hormones to regulate floral organ development. For example, auxin can coordinate with JA and GA to regulate stamen development in *Arabidopsis* [68]. Ethylene promotes the femaleness of cucumber and a 1-aminocyclopropane-1-carboxylic acid synthase CmACS11 was identified as sex determinant [71], whilst GA has a negative effect on female flower formation [72, 73]. As reported in spinach, GA can promote maleness and a *DELLA* gene *GIBBERELLIC ACID INSENSITIVE* (*GAI*) was defined as feminizing factor [39]. In this paper, 67 DEGs were related to hormones, including auxin, ethylene, GA, CK, ABA, JA, BR and SA (Additional file 5). All of them displayed sex-biased expression and only two auxin-related genes, *Spo12116* and *Spo22710*, were found to display male-specific expression (Fig. 6, Additional file 5). Hence, these two auxin-related genes can be served as candidates for studying the mechanism of spinach sex differentiation.

Transcription factors are pivotal factors for transporting endogenous and exogenous signals to regulate plant growth. Among the sex-biased genes, we found six male-specific transcription factors, including two NAC genes (*Spo02680* and *Spo17876*), two bHLH genes (*Spo26350* and *Spo26939*), one WRKY gene (*Spo09488*) and one C3H gene (*Spo24792*), and one female-specific transcription factor C2H2 gene (*Spo23846*) (Fig. 6). The male-specific NAC gene *Spo02680* located on sex chromosome chr04 (chr4: 88744812–88,747,497 bp) and its homologous gene in *Arabidopsis* is *SOMBRERO* (*AT1G79580*), regulating the cell fate during root cap development [74]. The homologous gene in *Arabidopsis* of another male-specific NAC gene *Spo17876* is *TAPNAC* (*AT1G61110*), a tapetal-specific *NAC* gene, affecting tapetum and pollen development and male fertility [75, 76]. The male-specific bHLH gene *Spo26350* locates on sex chromosome (chr4: 108742184–108,748,668 bp) and its homologous gene in *Arabidopsis* is *ABORTED MICROSPORES* (*AT2G16910*), regulating tapetal cell development and affecting male fertility and pollen differentiation [77]. The male-specific C3H gene *Spo24792* is homologous to *Arabidopsis* gene *CALLOSE DEFECTIVE MICROSPORE 1* (*AT1G68200*), which influences the male fertility by regulating callose metabolism during microspores development [78]. The female-specific C2H2 gene *Spo23846* is homologous to *Arabidopsis* gene *TRANSPARENT TESTA 1* (*AT1G34790*), which was expressed during ovules and seeds development [79]. Clearly, these gender-specific transcription factors may be associated with spinach sex differentiation and we will verify their function in our future work.

### Female and male network

Uncovering the mechanism of sex differentiation is helpful to identify sex-determining gene. We constructed the regulation network of spinach sex differentiation via WGCNA. Interestingly, we found two modules with sex-biased expression, i.e. female module and male module (Figs. 7; 8; 9).

In female network (Fig. 9; Table 2; Additional file 7), the hub gene is *Gene005140* (WRKY23) and its homologue in *Arabidopsis* is *AtWRKY23*, an auxin-responsive transcription factor and playing a role in auxin canalization [80]; moreover, overexpression of tomato *WRKY23* (homologue of *AtWRKY23*) in *Arabidopsis* altered the flowering time [81]; hence, we speculate that auxin may be the key hormone involved in female flower development. *AGO5* (*Spo10951*) was the only floral-related gene except hormone and TFs in female network. As reported, *AtAGO5* can interact with miR156 to regulate flowering by suppressing SPL transcription factors [82]. As shown in Fig. 9 and Additional file 7, *Spo24877* (*GAST1*) is the most closely correlated gene both of hub gene *WRKY23* and floral-related gene *AGO5* (weight value is 0.4491); and *Gene006949* (cyclin D3 protein) is the most closely correlated gene of *GAST1.* Hence, auxin may interact with GA to regulate spinach female flower development via *WRKY23* and cell cycle-related gene cyclin D3.

In male network (Fig. 9; Table 2; Additional file 7), the hub gene is *Gene000891* (*MES3*) and its most correlated gene is *Spo08103* (*GASA6*); the homologue in *Arabidopsis* of *MES3* is *AtMES3*, which participates in auxin pathway and can hydrolyze MeIAA to active IAA [83]. Furthermore, of the DEGs-related to hormone, two auxin-related genes, *Spo12116* and *Spo22710*, displayed male-specific expression (Fig. 6; Additional file 6). Hence, similar to female module, auxin may be the crucial hormone regulating male flower development. Except of TFs and the genes related to hormones, two UDP-glucose 4-epimerase family genes, both homologous to *Arabidopsis UGE1*, are involved in flower development. As reported, *AtUGE1* (AT1G12780) is involved in sugar flux and may be regulated by transcription factor HEME ACTIVATOR PROTEIN 3b to affect flowering [84, 85]. The closely correlated genes of spinach *UGE1* are *Spo08103* (*GASA6*) and *Gene005514* (*FBP2*) (Fig. 9; Additional file 7). Sugar signaling pathways can interact with various hormones to modulate plant growth. Daily fluctuations in GA sensitivity track the fluctuations in sugar levels and are regulated by the circadian clock [86, 87]. DELLA protein is an inhibitor of GA activity, whereas sucrose can stabilize DELLA proteins [87, 88], explaining the negative effect of GA on the sucrose-dependent induction of anthocyanin synthesis [89, 90] and indicating the involvement of DELLAs in the sucrose–GA interaction [91–93]. MADS-box family genes play important roles in flower development and form the ABCDE model to pattern floral whorls. The floral MADS-box proteins can target cell development-related genes to control flower development [94–97]. The cucumber *DELLA* gene *CsGAIP* may negatively regulate the B class floral homeotic genes *AP3* and *PI* to inhibit staminate development [98]. The B class floral identity genes, *AP3* and *PI*, act as masculinizing factors that participate in the establishment of sexual dimorphism in spinach [35, 36]. Hence, auxin, GA and sugar may cooperate together to regulate spinach male flower development through MADS-box transcription factor.

Comparing the co-expression network of female and male flower, we found auxin and GA were the common crucial factors in regulating female or male flower development; however, the co-expressed genes of these two factors were different, which may result in spinach sex differentiation. These hypotheses need to be verified in future work.

## Methods

### Plant materials and growth conditions

All plants used in this study were *Spinacia oleracea* L. cv DA JIAN YE BO CAI, a dioecious plant. Seeds were obtained from U.S. National Plant Germplasm System (https://npgsweb.ars-grin.gov/gringlobal/search.aspx?, accession number is PI 527332) and grown in an experimental field at Henan Normal University, Xinxiang, China (113.90°E, 35.32°N). Following the method of Sherry et al. [99], female flowers were collected when they were 0.3 mm in diameter (without stigma, FNS), 0.5 mm in diameter (without stigma, FNB), and mature with stigma (FYS). Male flowers were collected when they were 0.3 mm (M03), 0.5 mm (M05), and 1.0 mm (M10) in diameter. Flowers were sampled from five individuals at each stage and divided into three replicates. Female and male flowers collected at three stages were separately used for Illumina RNA-seq sequencing. The mixture of female and male flowers was used for PacBio Iso-seq sequencing.

### PacBio Iso-seq sequencing

Total RNA of the mixture of female and male flowers was prepared by TRIzol reagent (Life technologies). mRNA was enriched by Oligo (dT) magnetic beads. The enriched mRNA was reverse transcribed into cDNA using Clontech SMARTer PCR cDNA Synthesis Kit. PCR cycle optimization was performed to determine the optimal amplification cycle number for downstream large-scale PCR reactions. The optimized cycle number was then used to generate double-stranded cDNA. In addition, size selection of Cdna with > 4 kb was performed using the BluePippin TM Size-Selection System and mixed equally with no-size-selection cDNA. Large-scale PCR was performed for the next SMRTbell library construction. cDNAs were DNA damage repaired, end repaired, and ligated to sequencing adapters. The SMRTbell template was annealed to sequencing primer and bound to polymerase and sequenced on the PacBio Sequel platform using P6-C4 chemistry with 10 h movies by Gene Denovo Biotechnology Co. (Guangzhou, China).

### Annotation

The raw sequencing reads of cDNA libraries were classified and clustered into transcript consensus using the SMRT Link v5.0.1 pipeline [100] supported by Pacific Biosciences. The high-quality consensus sequences were then mapped to the reference genome using GMAP (version 2018-05-30) with (min_fl_count = 1, other parameters were default value) [101]. Redundant transcripts were collapsed with minimum identity of 95% and minimum coverage of 99%. The obtained isoforms were compared with reference genome (http://www.spinachbase.org/, spinach genome sequence_v1) [33] annotation and classified into three groups: known isoforms (mapped to annotated genes), new isoforms (mapped to different exons of annotated gene), and novel isoforms (mapped to unannotated region of reference genome).

To investigate the functions of new isoforms, we blasted new isoforms against the Nr database (http://www.ncbi.nlm.nih.gov), the Swiss-Prot protein database (http://www.expasy.ch/sprot), and the KEGG database (http://www.genome.jp/kegg) with the BLASTx program (http://www.ncbi.nlm.nih.gov/BLAST/, version 2.6.0+) at an E-value threshold of 1e− 5 to evaluate sequence similarity with genes of other species. GO annotation was analyzed by Blast2GO software (version 2.5.0) [102] with Nr annotation results of isoforms. Isoforms ranking the first 20 highest scores and no shorter than 33 high-scoring segment pair hits were selected to conduct Blast2GO analysis (default parameter). The functional classification of isoforms was performed using the WEGO software (version 2.0, default parameter) [103].

### Structural analyses

Alternative splicing events were identified using the SUPPA tool (version 2.2.1, default parameter) [104]. Seven major types of alternative splicing events, namely, ES, RI, A5, A3, MX, AF, and AL, were classified, counted, and compared between different samples.

Polyadenylation site was analyzed using all high-quality isoforms aligning to transcripts. The end position is the potential alternative polyadenylation site.

CNCI software (version 2) [105] and CPC software (http://cpc.cbi.pku.edu.cn/, version 0.9-r2) [106] were used to assess the protein-coding potential of novel and new isoforms by default parameters. Isoforms were mapped to the Swiss-Prot database to assess protein annotation. The intersection of both nonprotein-coding potential results and nonprotein annotation results were defined as lncRNAs. To further annotate lncRNAs at the evolutionary level, we used the software Infernal (http://eddylab.org/infernal/, version 1.1.1) [107] in sequence alignment. LncRNAs were classified by secondary structures and sequence conservation.

### Illumina RNA-seq sequencing and bioinformatics analyses

After the total RNA of male and female flowers was extracted by TRIzol reagent (Life technologies), mRNA was enriched by Oligo (dT) beads. The enriched mRNA was fragmented into short fragments using fragmentation buffer and reverse transcribed into cDNA with random primers. Second-strand cDNA was synthesized by DNA polymerase I, RNase H, dNTP, and buffer. The cDNA fragments were then purified with QiaQuick PCR extraction kit, end repaired, poly(A) added, and ligated to Illumina sequencing adapters. The ligation products were size selected by agarose gel electrophoresis, PCR amplified, and sequenced using Illumina HiSeq™ 2500 by Gene Denovo Biotechnology Co. (Guangzhou, China). After filtering, high-quality clean reads were assembled and mapped to the reference genome (http://www.spinachbase.org/) using TopHat2 (version 2.1.1) and Cufflinks (version 2.2.1) [108, 109].

Gene abundances were quantified by RSEM software (version 1.2.19) [110]. Gene expression level was normalized using the method of fragments per kilobase of transcript per million mapped reads. edgeR package (http://www.r-project.org/, version 3.12.1) was used to identify differentially expressed genes across samples or groups. Genes with a fold change of ≥2 and a false discovery rate (FDR) of < 0.05 in a comparison were designed as significant DEGs. DEGs were then subjected to enrichment analysis of GO functions and KEGG pathways.

The GATK (version 3.4–46) was used for calling variants of transcripts, and ANNOVAR (version 42,401) was used for SNP/InDel annotation. The function, genome site and type of variation of SNPs were also analyzed.

### Weighted gene co-expression network analysis

Co-expression networks were constructed using the WGCNA (v1.47) package in R (version 3.4.4) [111]. After filtering, gene expression values were imported into WGCNA to construct co-expression modules by using the automatic network construction function blockwiseModules with default settings, except that the power was 6, TOMType was unsigned, mergeCutHeight was 0.15, and minModuleSize was 50. Genes were clustered into 21 correlated modules.

To determine biologically significant modules, we used module eigengenes to calculate the correlation coefficient with samples. The intramodular connectivity (function softConnectivity) of each gene was calculated, and genes with high connectivity tended to be hub genes that may have important functions. The networks were visualized using Cytoscape_3.3.0.

GO and KEGG pathway enrichment analyses were conducted to determine the biological functions of genes in each module. Significantly enriched GO terms and pathways in genes in a module compared with the background were defined by hypergeometric test and a threshold of FDR of < 0.05.

### PCR and qPCR experiments

Total RNA was isolated using the TRIzol reagent (Invitrogen) according to the user’s manual. Approximately 1 μg of total RNA was used to synthesize the first-strand cDNA using PrimeScript™ RT reagent Kit with gDNA Eraser (TaKaRa). PCR validation of alternative spilcing, novel isoform, and lncRNA was performed by *TransStart*® *FastPfu* Fly DNA Polymerase (TRANSGEN). For alternative polyadenylation validation, we used 3′-Full RACE Core Set with PrimeScript™ RTase for cDNA synthesis and PCR amplification (TaKaRa). PCR product was monitored on 1% agarose gel. To validate the DEGs expression, qPCR was carried out with FastSYBR Mixture (CWBIO) on LightCycler® 480 System (Roche) according to the manufacturer’s instructions. UBQ was used as an internal control for normalization. The relative expression level of transcript was calculated with 2^−ΔΔC^_T_ method and the result was shown in Additional file 8. All primers sequences are listed in Additional file 9.

## Supplementary Information

**Additional file 1.** Flower tissue samples used in transcriptome sequencing.

**Additional file 2.** Validation of alternative splicing and alternative polyadenylation. (a) PCR validation of alternative splicing events (left) and gene structure (right); exons are represented by boxes, introns by lines; “ref” means gene structure identified in reference genome, “Sp” means gene structure identified by Iso-seq; “F” means forward primer, “R” means reverse primer. (b) Validation of polyadenylation sites by 3′ RACE PCR (left) and structure of the 3′ end (right); “□” represents exon, “─” represents intron”, “■” represents 3′ UTR; “—A(n)—” represents poly(A) structure; “●” represents the adaptor used in 3′ RACE; “F” means forward primer, “R” means reverse primer.

**Additional file 3.** PCR validation of novel genes and lncRNAs. “─” represents intron”, “■” represents exon, “F” means forward primer, “R” means reverse primer.

**Additional file 4.** Summary of RNA-seq data. (a) PCA analysis of all samples. (b) The top 20 enriched KEGG pathway. (c) GO Directed Acyclic Graph of DEGs between female and male flower at three early development stages in biological process. (d) GO Directed Acyclic Graph of DEGs between female and male flower at three early development stages in molecular function.

**Additional file 5.** List of sex-biased genes related to transcription factors, hormones, sugar and phenylpropanoid.

**Additional file 6.** Correlation between WGCNA module memberships.

**Additional file 7.** List of genes in female and male network.

**Additional file 8.** Expression validation of the DEGs by qPCR.

**Additional file 9.** Primer list used in this study.

**Additional file 10.** Summary of sex-linked genes with SNPs.

## Data Availability

Sequencing data that support the findings of this study have been deposited in the NCBI SRA database [https://www.ncbi.nlm.nih.gov/sra/PRJNA607896]. All data generated or analyzed during this study are included in this published article.

## Abbreviations

lncRNA: long noncoding RNA
WGCNA: Weighted gene co-expression network analysis
SDR: Sex-determining region
*GAI*: *GIBBERELLIC ACID INSENSITIVE*
*AP3*: *APETALLA3*
*PI*: *PISTILLATA*
KEGG: Kyoto Encyclopedia of Genes and Genomes
GO: Gene Ontology
Nr database: NCBI non-redundant protein database
HXK: Hexokinase
SE: Skipping exon events
A5: 5′ splice sites events
A3: 3′ splice sites events
MX: Mutually exclusive exons events
RI: Retained intron events
AF: Alternative first exons events
AL: Alternative last exons events
GA: Gibberellin
CK: Cytokinin
ABA: Abscisic acid
BR: Brassinosteroid
JA: Jasmonate
