# Supplementary figures and images for: Comparative transcriptome analysis of male and female flowers in Spinacia oleracea L
Source: BMC Genomics. 2020 Dec 1;21:850. doi: 10.1186/s12864-020-07277-4 (PMC7708156; doi:10.1186/s12864-020-07277-4)

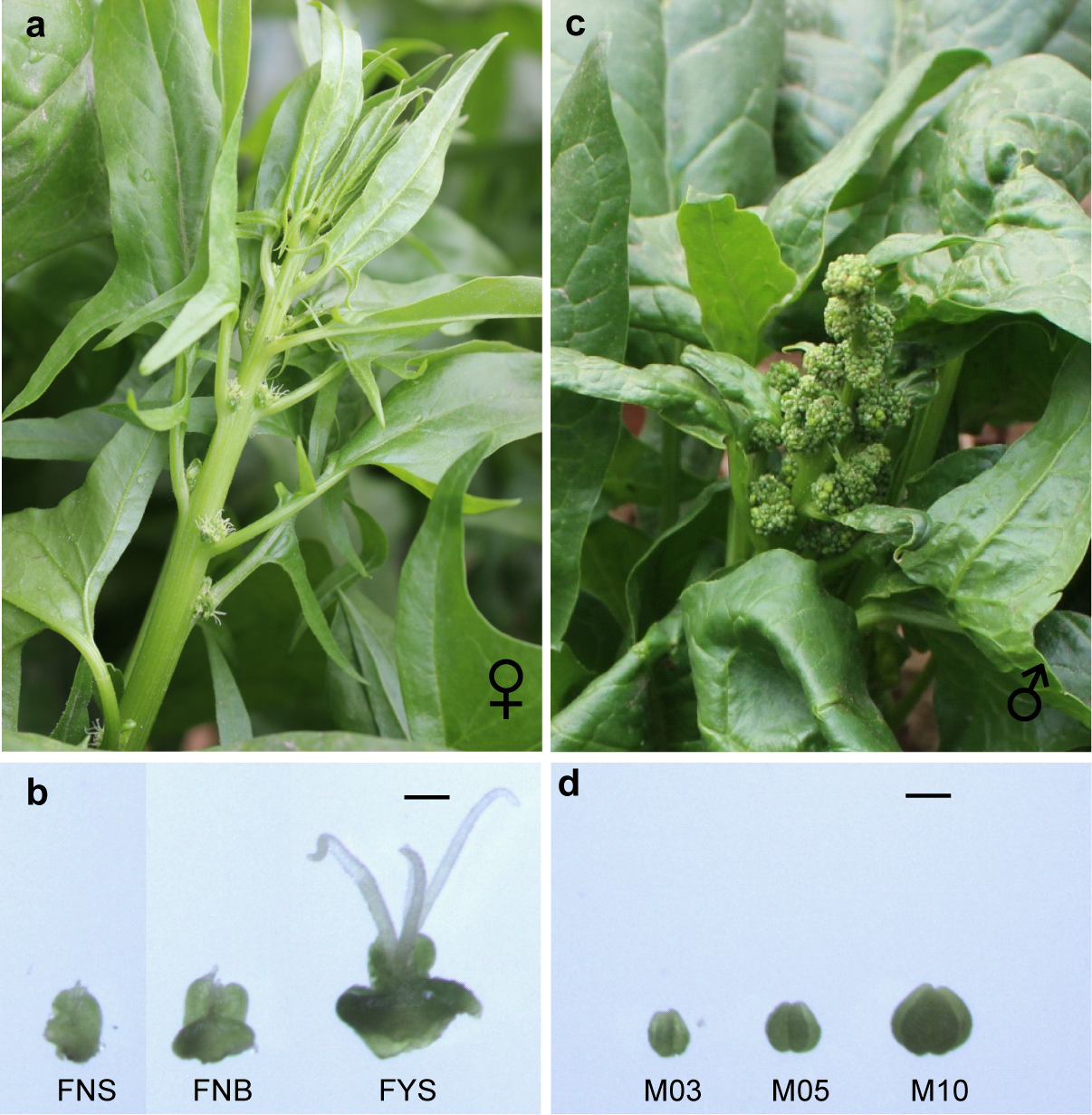

Supplement: Supplementary file 1 — Additional file 1. Flower tissue samples used in transcriptome sequencing. [file 12864_2020_7277_MOESM1_ESM.tif]

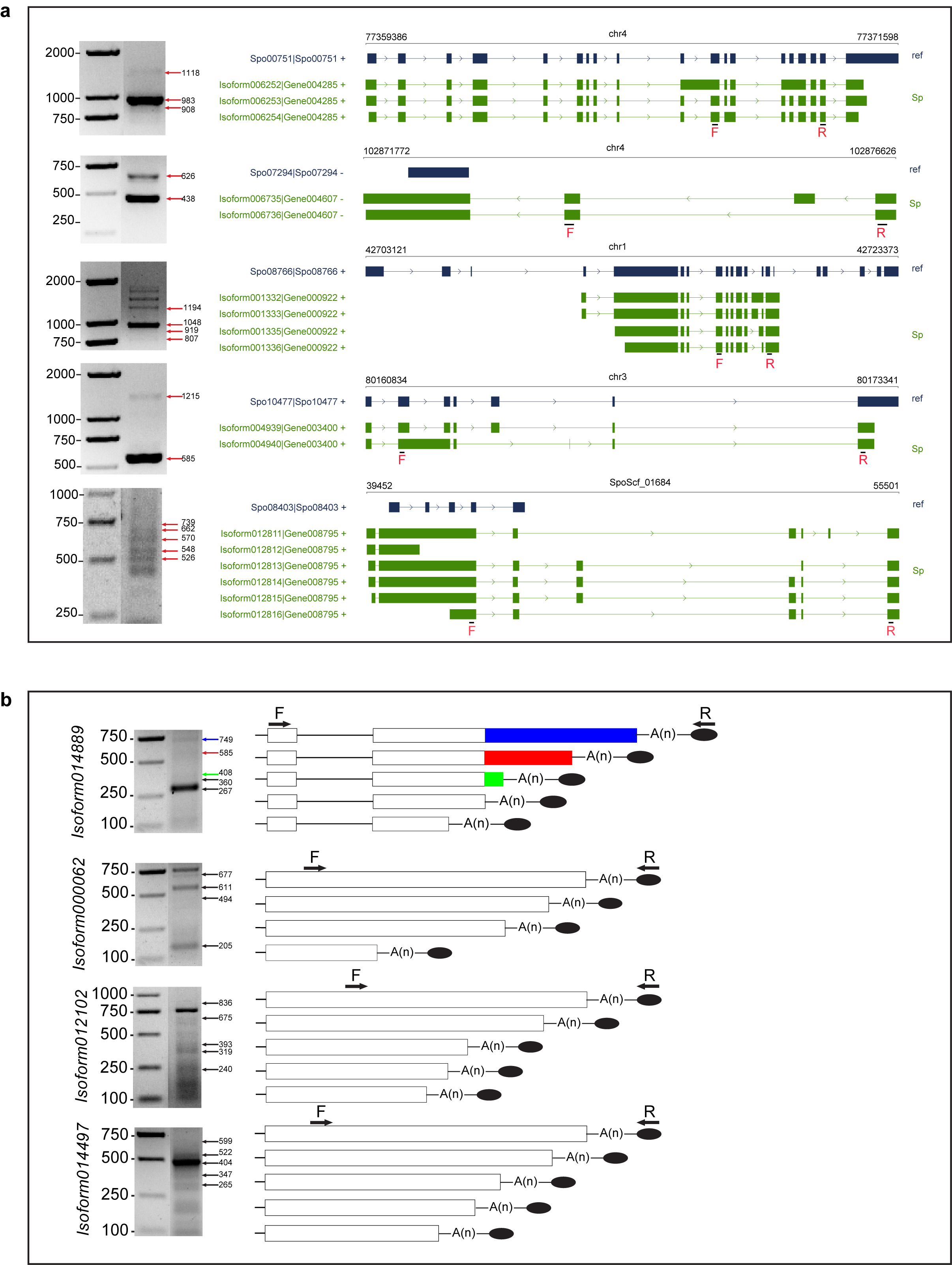

Supplement: Supplementary file 2 — Additional file 2. Validation of alternative splicing and alternative polyadenylation. (a) PCR validation of alternative splicing events (left) and gene structure (right); exons are represented by boxes, introns by lines; “ref” means gene structure identified in reference genome, “Sp” means gene structure identified by Iso-seq; “F” means forward primer, “R” means reverse primer. (b) Validation of polyadenylation sites by 3′ RACE PCR (left) and structure of the 3′ end (right); “□” represents exon, “─” represents intron”, “■” represents 3′ UTR; “—A(n)—” represents poly(A) structure; “●” represents the adaptor used in 3′ RACE; “F” means forward primer, “R” means reverse primer. [file 12864_2020_7277_MOESM2_ESM.tif]

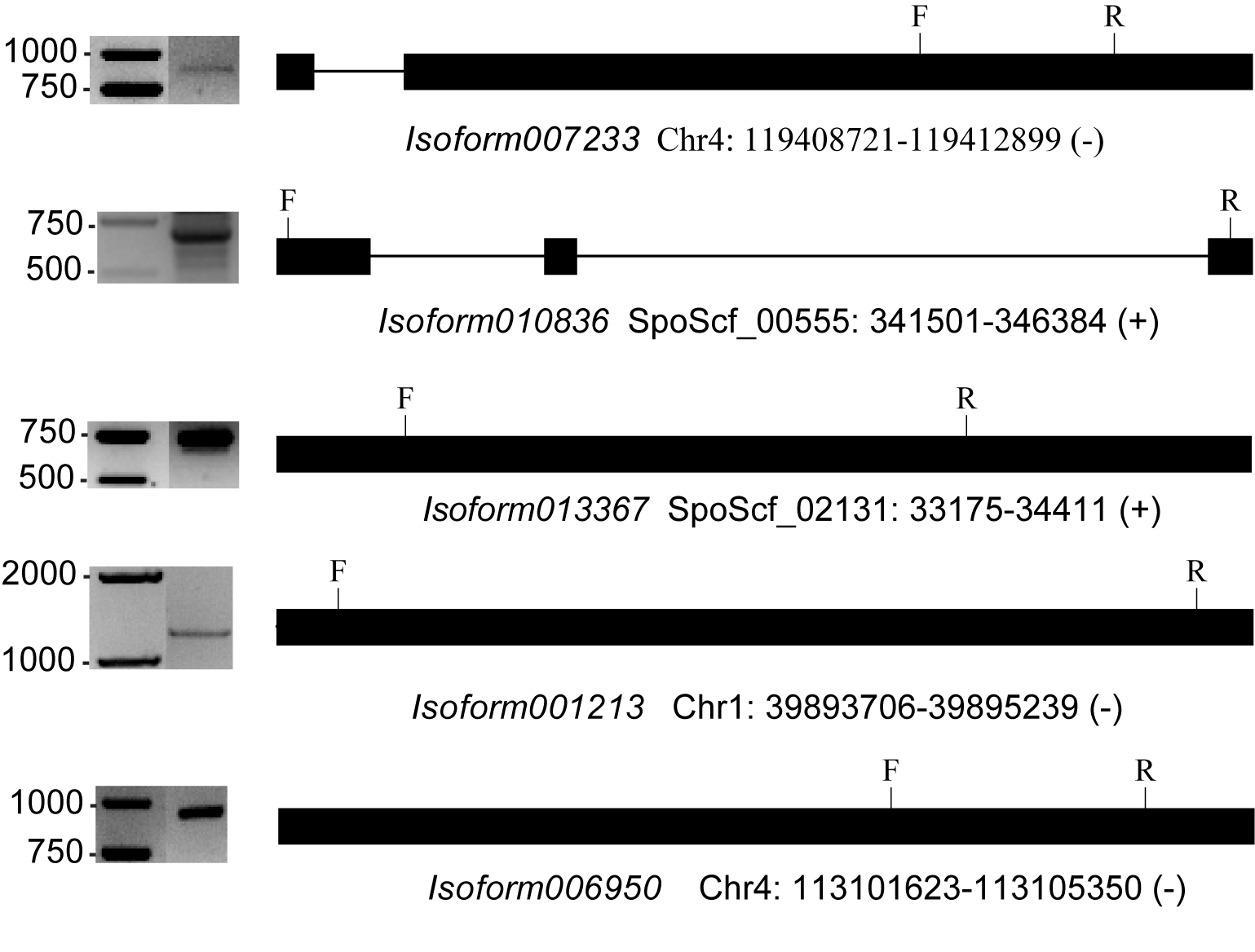

Supplement: Supplementary file 3 — Additional file 3. PCR validation of novel genes and lncRNAs. “─” represents intron”, “■” represents exon, “F” means forward primer, “R” means reverse primer. [file 12864_2020_7277_MOESM3_ESM.tif]

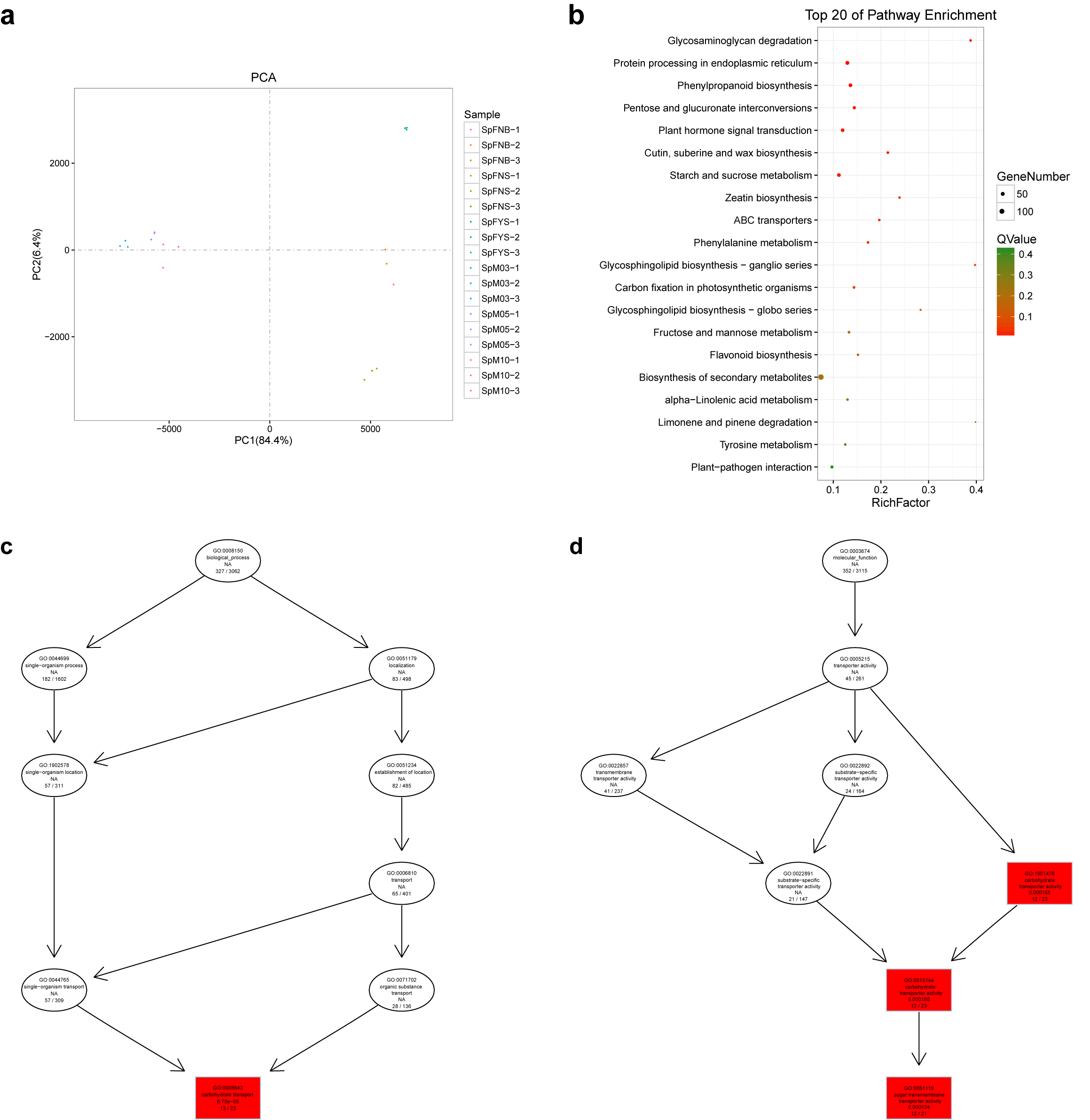

Supplement: Supplementary file 4 — Additional file 4. Summary of RNA-seq data. (a) PCA analysis of all samples. (b) The top 20 enriched KEGG pathway. (c) GO Directed Acyclic Graph of DEGs between female and male flower at three early development stages in biological process. (d) GO Directed Acyclic Graph of DEGs between female and male flower at three early development stages in molecular function. [file 12864_2020_7277_MOESM4_ESM.tif]

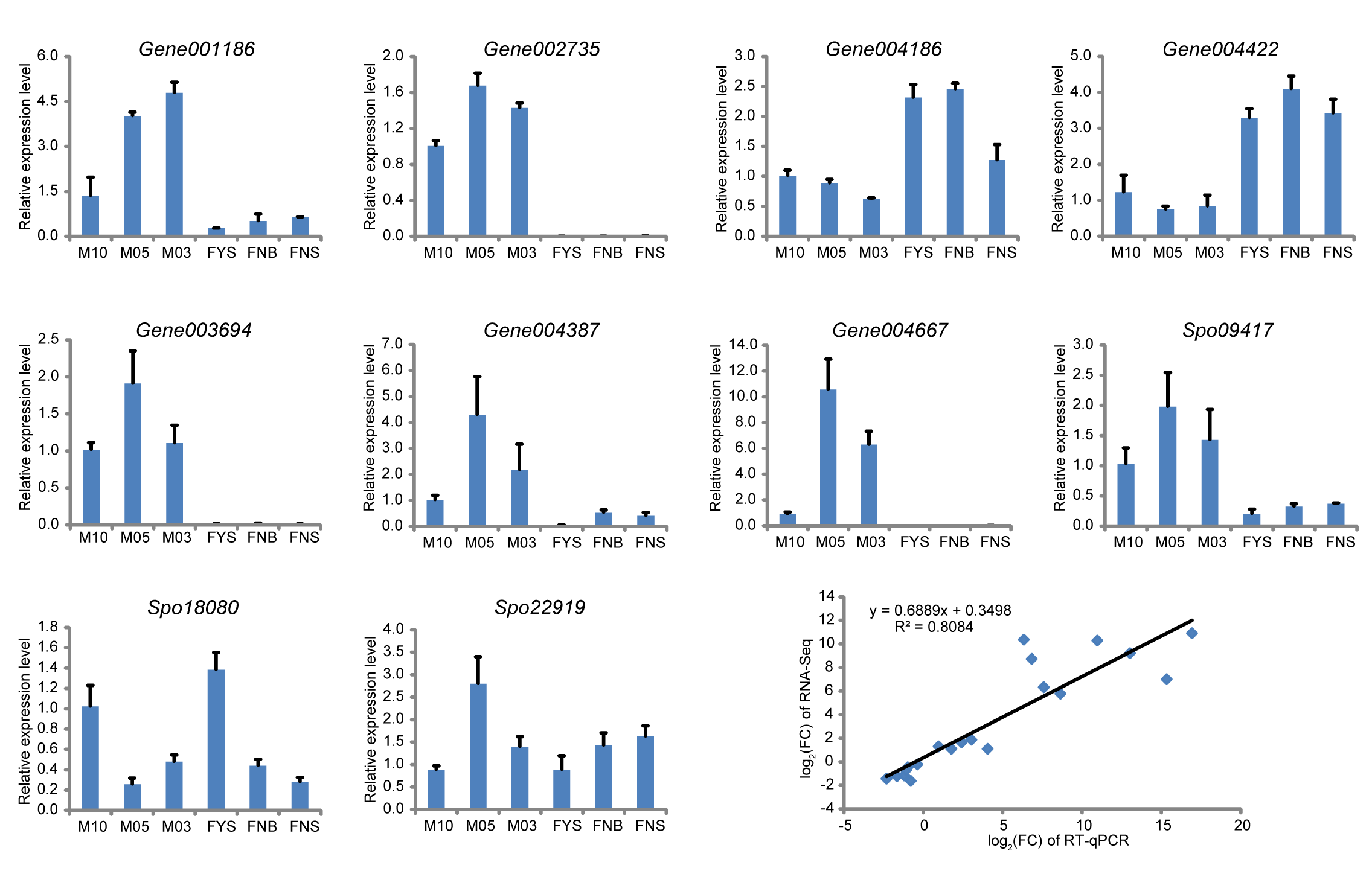

Supplement: Supplementary file 8 — Additional file 8. Expression validation of the DEGs by qPCR. [file 12864_2020_7277_MOESM8_ESM.tif]
